# Relative Contribution of Obesity, Sedentary Behaviors and Dietary Habits to Sleep Duration Among Kuwaiti Adolescents

**DOI:** 10.5539/gjhs.v8n1p107

**Published:** 2015-05-15

**Authors:** Ahmad A. Al-Haifi, Hana Th. AlMajed, Hazzaa M. Al-Hazzaa, Abdulrahman O. Musaiger, Mariam A. Arab, Rasha A. Hasan

**Affiliations:** 1 Food and Nutrition Science Department, College of Health Sciences, PAAET, Kuwait; 2 Applied Medical Sciences Department, College of Health Sciences, PAAET, Kuwait; 3 PediatricExercise Physiology Research Laboratory, King Saud University, Riyadh, Saudi Arabia; 4 Arab Center for Nutrition, Manama, Bahrain; 5 Physical Education Department, College of Basic Sciences, PAAET, Kuwait

**Keywords:** adolescents, eating habits, Kuwait, obesity, sedentary behaviours, sleep duration

## Abstract

The aim of this study was to investigate whether body mass index (BMI), eating habits and sedentary behaviours were associated with sleep duration among Kuwaiti adolescents. The study is part of the Arab Teens Lifestyle Study (ATLS), which is a school-based cross-sectional multi-center collaborative study. A sample of 906 adolescents (boys and girls) aged 14-19 years was randomly selected from 6 Kuwaiti Governances using a multistage stratified cluster sampling technique. The findings revealed that the prevalence of overweight and obesity was 50.5% in boys and 46.5% in girls. The majority of boys (76%) and of girls (74%) fell into the short sleep duration category (6 hours/day or less). Sleep duration were found to be negatively associated with BMI (girls only). Watching television (boys and girls) and working on computers (boys only) were also negatively associated with sleep duration. While the consumption of breakfast (both genders) and milk (boys only) was positively associated with sleep duration (*p*<0.05). In contrast, the consumption of fast foods (both genders), sugar-sweetened drinks and sweets (boys only) potatoes (girls only) were negatively associated with sleep duration (*p*<0.05). It can be concluded that the majority of Kuwaiti adolescents exhibit insufficient sleep duration which was associated with obesity measure, a combination of poor eating habits and more sedentary behaviors. The findings also suggest gender differences in these associations. Therefore, adequate sleep is an important modifiable risk factor to prevent obesity and was positively associated with some unhealthy lifestyle habits.

## 1. Introduction

Obesity in children and adolescents is an increasing worldwide health problem on both physical and psychological health (UN, 2008). Moreover, studies have shown that about 70% of obese adolescents grow up to become obese adults (Nicklas, Baranowski, Cullen, & Berenson 2001). The prevalence of obesity in Kuwait is among the highest in this region (Ministry of Health, 2010; Abdul-Rasoul, 2012; Musaiger et al., 2012; Abdulnabi et al., 2012). Despite considerable attempts to control obesity worldwide in recent years, its prevalence has raised dramatically, especially among Kuwaitis adolescents (Al-Haifi et al., 2013).

The high prevalence of obesity among Kuwaiti adolescents is largely due to the rapid socio-economic growth, including changing in eating habits, which characterized by the availability and consumption of fast food, irregular meal pattern, skipping breakfast, decreased intakes of fibre, and increase intakes of sweets and sugars (Musaiger et al., 2013; Al-Haifi et al., 2013; Zaghloul et al., 2013). In addition, a lower time spent in physical activity compared to that spent in sedentary activities, such as television viewing, and video and computer games (Al-Haifi et al., 2013; Kilani, Al-Hazzaa, & Waly, 2013). Therefore, it is likely that any relationship between poor eating habits, physical activity and body mass index (BMI) is moderated through other lifestyle behaviors.

Adequate sleep has been considered important for the adolescent’s biological and psychological health (Chen, Wang, & Jeng, 2006; Dahl & Lewin, 2002). Adolescents obtaining adequate sleep were shown to have higher frequency of health-promoting behaviors (Chen, Wang, & Jeng, 2006; Al-Hazzaa, Musaiger, Abahussain, Al-Sobayel, & Qahwaji, 2014). On the other hand, sleep restriction can have adverse effects on endocrine function, cognitive performance, and metabolic as well as inflammatory responses (Banks & Dinges, 2007).

Critical reviews of the epidemiological evidence indicated that short sleep duration appears independently associated with weight gain but most of the available studies have diverse consistency (Chen, Beydoun, & Wang, 2008; Marshall, Glozier, & Grunstein 2008; Patel & Hu, 2008). Despite the low energy cost of sleep (less than1.0 calorie/hour per kg of body massin adults), population studies have shown that a frequent insufficient sleep duration is associated with excess body mass (Al-Hazzaa, Musaiger, Abahussain, Al-Sobayel, & Qahwaji, 2012; Taheri, Lin, Austin, Young, & Mignot, 2004; Gangwisch, Malaspina, Boden-Albala, & Heymsfield, 2005; Patel, Malhotra, White, Gottlieb, & Hu, 2006).

To the best of our knowledge, there are no studies attempted to assess the outcome of sleep duration on obesity among adolescents in Kuwait. Hence, the overall objective of the present study was to determine whether BMI, eating habits, physical activity and sedentary behaviors are associated with sleep duration among Kuwaiti adolescents.

## 2. Methods

The present study is part of the Arab Teens Lifestyle Study (ATLS). ATLS is a school-based cross-sectional multi-center collaborative study (Al-Hazzaa, Musaiger, & ATLS Group, 2011). The study protocol was approved by the Public Authority for Applied Education and Training as well as by the Ministry of Education and schools principals in Kuwait. Informed consent was obtained from the parent of each participant. Data were collected by trained researchers under the supervision of the lead author.

### 2.1 Participants

The study population represented adolescent males and females in Kuwait secondary schools enrolled in the school year 2009/2010. Sampling was based on multistage stratified random sampling technique. In the first stage, a systematic random sampling procedure was used to select the schools. The schools were stratified into boys and girls secondary schools, with further stratification into public and private schools. The selection of the private/public schools was done proportional to each one size. At the second stage, classes were selected at each grade level using a simple random sampling design. One class was randomly selected in each grade of the three grades (grades 10, 11 and 12) in each secondary school. Data were collected from all six Governances. Two schools from each Governance were chosen (one for boys and one for girls), then one class from each grade was randomly selected. A total of 36 classes plus 6 classes from a private school were selected, which included an average of 25 students in each class. All students in the selected classes, who were free from any physical deformity, were invited to participate in the study. The total number of selected students was 906 (463 males and 443 females).

### 2.2 Anthropometric Measurements

Anthropometric variables included body weight, height and waist circumference. All measurements were performed in the morning. Body weight was measured to the nearest 100 gram using a calibrated portable scale (Detecto scale, USA). Measurements were done with minimal clothing and without shoes. Height was measured to the nearest cm while the subject was in full standing position without shoes using calibrated measuring rod (Detecto scale, USA). Body mass index (BMI) was calculated as a ratio of weight in kg by height squared in meter. The International Obesity Task Force (IOTF) age and sex specific BMI cut-off reference standard (Cole, Bellizzi, Flegal, & Dietz, 2000) were used to identify overweight and obesity in adolescents between the ages of 14 and 17 years. Participants who were18 years and above, the WHO adults’ cut-points (WHO, 2000) was used. Waist circumference (WC) was measured horizontally to the nearest 0.1 cm using a non-stretchable measuring tape at the level of umbilical. Participants were measured in private at an examination cite in each school. Waist-height ratio (WHtR) was calculated as the ration of waist circumference in centimetres to height in centimeters.

### 2.3 Physical Activity Assessment

A validated self-reported questionnaire was used as the means to assess the level of physical activity of the young participants (Al-Hazzaa, Al-Sobayel, & Musaiger, 2011a; Al-Hazzaa, Abahussain, Al-Sobayel, Qahwaji, & Musaiger, 2011b). The questionnaire collects information on frequency, duration and intensity of a variety of light-, moderate- and vigorous-intensity physical activities during a typical week. The physical-activity questionnaire covered such domains as transport as well as household, fitness and sports activities. Physical activities were assigned metabolic equivalent (MET) values according to the compendium of physical activity (Ainsworth et al., 2011) and the compendium of physical activity for youth (Ridley, Ainsworth, & Olds, 2008). Moderate-intensity physical activity such as normal-pace walking, brisk walking, swimming, household activities and moderate-intensity sports, like volleyball, badminton and table tennis were included. Household activities were given a mean MET value of 3. Moderate-intensity recreational sports were assigned an average MET value equivalent of 4 METs. Slow walking, normal-pace walking and brisk walking were assigned MET values of 2.8, 3.5 and 4.5 METs, respectively, according to modified METs values from the compendium of physical activity for youth (Ridley et al., 2008). Further, vigorous-intensity physical activity and sports such as stair-climbing, jogging and running, cycling, self-defence, weight training and vigorous sports, like soccer, basketball, handball and singles tennis were also counted. Vigorous-intensity sports were assigned an average MET value equivalent of 8 METs. The percentage of adolescents who met daily physical activity recommendations (Tremblay et al., 2011) was calculated, using cut-off scores equivalent to one hour per day of moderate-intensity (4 METs) physical activity. This amount of exercise duration was then converted into METs-min per week that is corresponding to 1680 METs-min/week (60 min per day x 7 days per week x 4 METs).

### 2.4 Sedentary Behaviours

Sedentary behaviours questions included the time spent on television (TV) viewing, video and computer games as well asinternet use. Participants were asked to provide the average number of daily hours spent in such activity without differentiating between weekdays and weekends. For the total screen time cut-off points, we used the American Academy of Paediatrics guidelines of a maximum of 2 hours per day for sedentary behaviors (American Academy of Pediatrics, 2001). Sleep duration was categorized into two categories; below or 7 hours or more per day.

### 2.5 Eating Habits

In addition to the physical-activity questionnaire, the ATLS questionnaire included some questions related to the frequency of certain dietary habits of adolescents. The questions asked how many times per a typical week the participants consumed breakfast, sugar-sweetened drinks including soft beverages, vegetables (cooked and uncooked), fruit, milk and dairy products, cakes and donuts, chocolate and candy, fast foods and energy drinks. The fast foods in this regard included examples from both Western fast foods and Arabic fast-foods. These questions covered healthy and unhealthy dietary habits. The students had to choice from zero intake to a maximum intake of 7 days per week (every day). For the dietary cut-off points, we calculated the proportions of adolescents who had a daily intake of breakfast, fruit, vegetables and milk and those participants who exceeded 3 days’ intake per week of the unhealthy dietary habits, such as sugar-sweetened drinks or fast foods.

### 2.6 Data Analysis

Data analysis was performed on a personal computer with the statisticalsoftware package SPSS version 17.0. Descriptive statistics were performed on all the variables and were reported as mean values and standard deviations or percentages. In addition, General Linear Model (GLM) was used to determinethe variability in sleep duration that is attributable to physical activity, sedentary behaviours and eating habits. GLM method allows regression analysis to be carried out in the presence of fixed factors such as categories of physical activity. It also allows activity, sedentary behaviours and eating habits to be analyzed as continuous variables. The partial eta squared was used in the present study, which represents the variance due to the effect variable plus error (Kirk, Mutrie, MacIntyre, & Fisher, 1982). Variance was excluded due to the independent effects of other variables. Partial eta squared provides the contribution of each variable or interaction as if it was the only variable, so it is not masked by other variables. In the current study, partial eta squared was used as a measure of effect size (proportion of the variance in the sleep duration, which is attributable to physical activity, sedentary behaviours and eating habits). A *P* valueof less than 0.05 was considered statistically significant.

## 3. Results

Table 1 represents the characteristics of the participants. There was no significant difference (*p* = 0.137) between boys and girls in the mean of the sleep duration (boys: 5.2 ±1.6 vs. girls: 5.1 ±1.7). In addition, there were no significant differences between boys and girls in mean age (*p* =0.365), BMI (*p* = 0.113) or WC (*p* = 0.074). However, mean differences relative to sex were significant in body weight (*p* < 0.001), height (*p* < 0.001) and WHtR (*p* < 0.001). Table 2 shows the majority of boys (74.2%) and of girls (76.1%) fell into the short sleep duration category (less than 7 hours/day).

**Table 1:** The anthropometric characteristics of the participants*

	Boys (n= 463)	Girls (n= 443)	*P- value*
Age (year)	16.3 ± 1.1 (463)	16.2 ± 1.2 (443)	0.365
Weight (kg)	72.8 ± 21.0 (463)	62.6 ± 16.0 (442)	0.001
Height (cm)	169.3 ± 6.6 (463)	157.5 ± 6.0 (442)	0.001
BMI (wt/ht²) ^1^	25.3 ± 6.6 (463)	25.2 ± 6.0 (442)	0.113
Waist circumference (cm)	83.1 ± 16.4 (462)	84.9 ± 14.6 (437)	0.074
Waist circumference/ height	49.1 ± 9.3 (462)	53.9 ± 9.0 (437)	0.001
Sleep duration	5.2 ± 1.6 (461)	5.1 ± 1.7 (438)	0.137

* Data are means ± SD.

**Table 2:** Prevalence (%) of sleep duration categories among adolescents

Sleep duration categories	Boys (%)	Girls (%)
Short duration ^1^	76.1	74.2
Long duration ^2^	23.9	25.8
**Total N**	**461**	**438**

1 Short duration: equal or less than 6 hours/day;

2 Long duration: equal or more than 7 hours/day.

The proportions of variance for body weight, physical activity, sedentary behaviors and eating habits, explained by sleep durationare presented in Table 3. Among girls, sleep duration was significantly associated with BMI (*p* < 0.05), but not with WC and WHtR. For example, girls with normal ideal BMI have more sleep duration (5.1 hours/day) than those girls in obese category (4.8 hours/day) but there was no significant association in boys.

**Table 3:** The proportions of variance for body weight, physical activity, sedentary behaviors and eating habits, explained by sleep duration*

Independent Variables	Boys		Girls	
	
	*P-value*^1^	PES^2^	*P-value^1^*	PES^2^
Body mass index	0.193	0.004	0.029	0.011
Waist circumference	0.392	0.002	0.663	0.000
Waist/height ratio	0.362	0.002	0.885	0.000
Moderate activities^3^	0.080	0.007	0.214	0.004
Vigorous activities^4^	0.853	0.000	0.351	0.002
At least moderate activities^5^	0.428	0.001	0.262	0.003
TV hours	0.000	0.029	0.001	0.026
Computer hours	0.002	0.020	0.362	0.002
Vegetables	0.131	0.005	0.800	0.000
Fruits	0.453	0.001	0.180	0.004
Milk	0.039	0.009	0.876	0.000
Fast foods	0.034	0.010	0.007	0.017
Potatoes	0.370	0.002	0.009	0.016
Cakes/Donuts	0.054	0.008	0.173	0.004
Sweets	0.022	0.011	0.346	0.002
Sugar-sweetened drinks	0.001	0.024	0.365	0.002
Energy drinks	0.055	0.008	0.793	0.000
Breakfast	0.024	0.011	0.050	0.009

* P values and partial eta squared are shown for analysis using physical activity, sedentary behaviours and eating habits as continuous variables, without any adjustments.

1 P value: The mean difference is significant at the 0.05 level or less.

2 PES: partial eta squared (proportions of variance).

3 Moderate activities include activities such as normal pace walking, brisk walking, recreational swimming, badminton, table tennis.

4 Vigorous activities include activities such as jogging, running, self-defence, weight training, soccer, basketball.

5 At least moderate activities include both moderate and vigorous activities.

According to physical activity and sedentary behaviors, in boys, sleep duration was significantly associated with the number of times spent in watching TV and using computer (*p* < 0.05). Boys with who sleep an average 5.3 hours/day have more time spent in watching TV than those with sleep duration of 5.0 hours/day. In girls, sleep duration was only significant associated with the number of times spent in watching TV (*p* < 0.05). Other physical activities were not significant in both genders.

The intakes of milk, fast foods, sweet and sugar drinks were significant (*p* < 0.05) associated with sleep duration in boys. While in girls, only the intakes of fast foods and potatoes were significant (*p* < 0.05) associated with sleep duration. As for eating habits, sleep duration was also inversely related to breakfast intake in both genders (*p* < 0.05). Subjects who rarely ate breakfast have less sleep duration. The intake of other variables was not significantly associated with sleep duration in both gender.

In general, the proportions of variance (expressed in partial eta square) in the anthropometric data, eating habits, sedentary behaviors explained by sleep duration were found to be less than 2.9% in boys and less than 2.6% in girls. We have tried logistic regression analyses of the data and the results did not change much from the current findings.

## 4. Discussion

This study demonstrates a correlation between short sleep duration and BMI, dietary and eating habits and sedentary behaviours among Kuwaiti adolescents. There were significant differences between boys and girls. About three quarters 76.0% of boys and 74% of girls Kuwaiti adolescents did not obtain enough daily sleep, as sleep duration was equal or less than 6 hours per day. The higher proportion of hours in sleep duration, in the current study, appears to be lower compared to other studies. A report among Australian adolescents, age ranging from 15.5 to 17.5 year old, showed average sleep duration of 8.40 to 9.10 hours/day (Olds, Maher, Blunden, & Matricciani, 2010). Another study showed an average 8.35 hours of sleep/day among Spanish adolescents (Ortega et al., 2010). Finally, adolescents from 10 different European cities reported an average of 8 hours of sleep/day (Garaulet et al., 2011). However, sleep duration obtained in the present study was not much far from data from a previous study conducted on Saudi children, aged 6- to 13-years old, that reported shorter sleep duration compared with that found for children from Western countries (BaHammam, Bin Saeed, Al-Faris, & Shaikh, 2006).

The findings of the present study showed that about one third of the Kuwaiti adolescents did not obtain 7 hours of daily sleep, while about 75% of the participants had short duration of daily sleep (equal or less than 6 hour/day). In comparison with our findings, a recent study showed that about one third of the Saudi adolescents did not obtain 7 hours of daily sleep, while about 50% of the participants had less than 8 hours of daily (Bahammam, Al-Khairy, & Al-Taweel, 2005). Another study have reported insufficient sleep (<8 hours/day) on average school day by 68.9% of high school students in the United States (McKnight-Eily et al., 2011).

Furthermore, McKnight-Eily et al., (2011) reported that the proportions of adolescent boys and girls who reported less than 6 hours of daily sleep were 28.7% and 32.6%, respectively (McKnight-Eily et al., 2011) (10). Finally, among Taiwanese adolescents, 54% have reported that they slept less than the suggested 6 to 8 hours per day (Chen et al., 2006).

In the present study, many factors may be related to the short sleep duration among Kuwaiti adolescents. Adolescence is an important stage where there is a great change in the physiological, social and behavioral pattern that has a large influence on the adolescents life. In growing countries, such as Kuwait, the fast and easy daily lifestyle, availability of home- fast internet connections, multi-channeled TV programs; may contribute to the short sleep duration among adolescents. In addition, the availability of mobiles, I-pads and other electronic devices leads to the prevention of adolescents to sleep early. Adding to that is the large amount of daily school home-works as requested by our governmental schools and also parents demands to get higher scores in schools. Finally, Kuwait school system starts the school day in the early morning (around 7 am). All of that would place the adolescents at greater risk for sleep limitation. Previous research has indicated that early starting of school negatively affect total sleep time among school children (Ohida et al., 2004).

Some studies clearly demonstrated that short sleep duration is negatively related to weight gain while other studies in adults regularly showed an association of short sleep duration with obesity. But some studies demonstrated an association of long sleep duration with BMI. Conversely, some studies showed no association between long or short sleep with obesity (Chen, Beydoun, & Wang, 2008; Marshall, Glozier, & Grunstein 2008; Patel & Hu, 2008; Van Cauter & Knutson, 2008; Calamaro et al., 2010). However, a clear pattern where shorter sleep durations were associated with obesity among infants (Touchette et al., 2008; Taveras, Rifas-Shiman, Oken, Gunderson, & Gillman, 2008).

In the current study, short sleep duration among girls was significantly associated with BMI but no correlation was found among boys. Regional study involving school children aged 10 to 19 years from Riyadh has also shown that short sleep duration has significantly increases the risk of obesity in both boys and girls (Bawazeer et al., 2009). Among European adolescents, short sleep duration was shown to be associated with higher adiposity markers, particularly in females (Garaulet et al., 2011). Similar findings were also reported by studies conducted on Indian (Gupta et al., 2008) and Japanese adolescents (Sekine et al., 2002). In contrast to our findings, Huang et al. found no association between BMI and weekday total sleep time among Taiwanese adolescents (Huang, Wang, & Guilleminault, 2010).

Regarding eating habits, the present study proved that sleep duration was inversely related to breakfast intake in both genders. This is in accordance with a regional study in Muscat, Sultanate of Oman, where skipping breakfast was dominant among the Omani adolescence (Kilani et al., 2013). Recent study showed that skipping breakfast was significantly associated with short sleep duration among adult males (Nishiura, Noguchi, & Hashimoto, 2010). In contrast, T. Zilberter and E. U. Zilberter showed that breakfast is just a meal and not the most important meal in the day (T. Zilberter and E. U. Zilberter, 2014).

Our study showed that the intakes of milk, fast foods, sweet and sugar drinks were significantly associated with sleep duration in boys only, while in girls, only the intakes of fast foods and potatoes were significantly associated with sleep duration. Preference to fast food, sweetened drinks, sweets and potatoes is a global unhealthy eating habit among young adults and school ages students (WHO, 2004). Kuwait is not an exception, as food consumption patterns had changed dramatically since the past four decades, and the fat rich diet, sweet and sugar drinks are becoming in great demand among children and adolescents (Musaiger, 2011).

In the present study the intake of fruits and vegetables among boys and girls was not significant with sleep duration. This is accordance with a previous study that reported carbohydrate and fat intakes were higher for adolescents in Saudi Arabia than the consumption of fruits and vegetables (Al-Hazzaa, Abahussain, Al-Sobayel, Qahwaji, & Musaiger, 2011; Washi & Ageib, 2010).

In contrast, a recent study among Omani adolescents showed that the consumption of fruits and vegetables was good among both genders (Kilani et al., 2013). Also a recent study reported that adolescents who eat more fruit and vegetables have more sleep duration (Garaulet et al., 2011).

Our data showed that short sleep duration among Kuwaiti adolescents who were more sedentary and spent more time watching TV in both genders while playing computer games was only among boys. Many regional studies were in accordance with our data (Kilani et al., 2013; Al-Hazzaa et al., 2011; Washi & Ageib, 2010).

Recently, data of a systemic review demonstrated that sedentary behavior, usually measured as TV watching and computer game playing, is mainly associated with unhealthy dietary habits in adolescents (Pearson & Biddle, 2011). Regionally, in a study conducted on Iranian adolescents, it was shown that the most active adolescents consumed fruit, vegetables and dairy products more frequently than their less active peers (Kelishadi et al., 2007).

Also previous studies demonstrated that there is a correlation between sedentary behavior and eating habits, as intake of fruits and vegetables was significant in both genders (Collison et al., 2010). In French adolescents, it was reported that physical activity positively correlated with the consumption of fruit and vegetables (Platat et al., 2006). In addition, Kremers et al. showed that a low frequency of fruit consumption was associated with low physical activity among Dutch adolescents (Kremers, De Bruijn, Schaalma, & Brug, 2004).

Our study showed no significant association between sleep duration and physical activity and sedentary behaviors in both genders. This is also in agreement with other studies (Garaulet et al., 2011; Tu, Watts, & Masse, 2015; Foti, Eaton, Lowry, & McKnight-Ely, 2011; Olds, Maher, & Matricciani, 2011). Furthermore, our study showed that neither moderate nor vigorous activities nor at least moderate activity were related to sleep duration. This reduced activity pattern among Kuwaiti adolescents was significantly associated with the number of times spend in watching TV and playing computer games. Another mean reason for such sedentary life is due to the fact that physical activity lesson in Kuwait school system is very minimum (only once every week for no more than 45 minutes). Boys may be more active and easy going out while the girls, for social reasons, most of the day stayed at home. Another major factor which contributes to sedentary life among Kuwaiti adolescents is the great dependence on using cars rather than walking to school or supermarket and back due to the hot climate conditions. Society as well as parents has an essential role to minimize the time of watching TV and retiring to bed for their children and adolescents.

The present study has a number of limitations. First, this is a cross-sectional study and therefore causation cannot be inferred. Second, the data on sleep duration, physical activity and diet were collected using self-reported questionnaire which might have introduced some errors in estimation. Second limitation regarding dietary information, the consumption each of the selected foods was collected based on frequency per week without portion size. Nevertheless, the inclusion of representative sample of Kuwaiti adolescent with nearly 1000 participants makes it unlikely that the true situation will be different. Further strength of this study was that it used a validated questionnaire to assess anthropometric measurements and physical activity levels (employed metabolic equivalent to calculate energy expenditure from physical activity).

## 5. Conclusion

The present study observed a high prevalence of short sleep duration among Kuwaiti adolescents. The high prevalence of sedentary behaviors, physical inactivity and unhealthy dietary habits among Kuwaiti adolescents is a major public health concern. To the best of our knowledge, this is in fact the first study to be conducted in Arab adolescents, and not just in Kuwait. There is an urgent need for national policy promoting active living and healthy eating and reducing sedentary behaviors among children and adolescents in Kuwait and other Arab countries. Future interventions should investigate whether adopting a healthy lifestyle by adolescents with short sleep duration would improve their sleeping habits or not.
